# Macrophage Functions in Tissue Patterning and Disease: New Insights from the Fly

**DOI:** 10.1016/j.devcel.2017.01.001

**Published:** 2017-02-06

**Authors:** Will Wood, Paul Martin

**Affiliations:** 1 Department of Cellular and Molecular Medicine, Biomedical Sciences, University of Bristol, Bristol BS8 1TD, UK; 2 Departments of Biochemistry and Physiology, Pharmacology and Neuroscience, Biomedical Sciences, University of Bristol, Bristol BS8 1TD, UK; 3 School of Medicine, Cardiff University, Cardiff CF14 4XN, UK; 4 Lee Kong Chiang School of Medicine, Nanyang Technological University, Singapore 636921, Singapore

**Keywords:** *Drosophila*, macrophage, inflammation, apoptosis, development, wound, disease, migration, immunity

## Abstract

Macrophages are multifunctional innate immune cells that seed all tissues within the body and play disparate roles throughout development and in adult tissues, both in health and disease. Their complex developmental origins and many of their functions are being deciphered in mammalian tissues, but opportunities for live imaging and the genetic tractability of *Drosophila* are offering complementary insights into how these fascinating cells integrate a multitude of guidance cues to fulfill their many tasks and migrate to distant sites to either direct developmental patterning or raise an inflammatory response.

## Teaser

Wood and Martin discuss the similarities between mouse and *Drosophila* macrophages in the contexts of development, phagocytosis, patterning and immune response, and disease states. They discuss the advances allowed by live imaging and fly genetics, and how insights from both insect and mammalian systems can be combined.

## Main Text

### Introduction

Macrophage is a term first coined by Metchnikoff in the late 1800s to describe “big eating” cells that he observed in starfish embryos as they exhibited a foreign body response after he poked the embryos with a rose thorn (Metchnikoff, 1968). Macrophage-like cells exist in organisms from echinoderms to man, and besides their clear role as “professional” phagocytes, they appear, at least in higher organisms, to fulfill numerous other functions in almost all tissues, from the earliest developmental stages when they are first born in the embryo, through to adulthood where they both help maintain tissue homeostasis and have pivotal roles in the healthy and unhealthy inflammatory response to wounding and other tissue insults including cancer. These multiple roles for macrophages are exceedingly complex and are currently the target of considerable research. New studies in the genetically tractable *Drosophila* embryo, larvae, and pupae are offering useful additional insight into molecular mechanisms, particularly those underpinning how macrophages migrate within tissues and how they integrate several incoming cues to determine their responsive behavior in various circumstances. In this review we briefly describe what is known about the origins of mammalian macrophages and their functions in both developmental patterning of the embryo and during tissue repair, where it seems that embryonic morphogenesis is recapitulated to help rebuild damaged tissues. As some aspects of macrophage function and signaling are not yet tractable in mammals, here we describe *Drosophila* studies that might help fill the gaps and guide the way forward.

### Origins of Mammalian Macrophage Lineages

**Figure 1:**
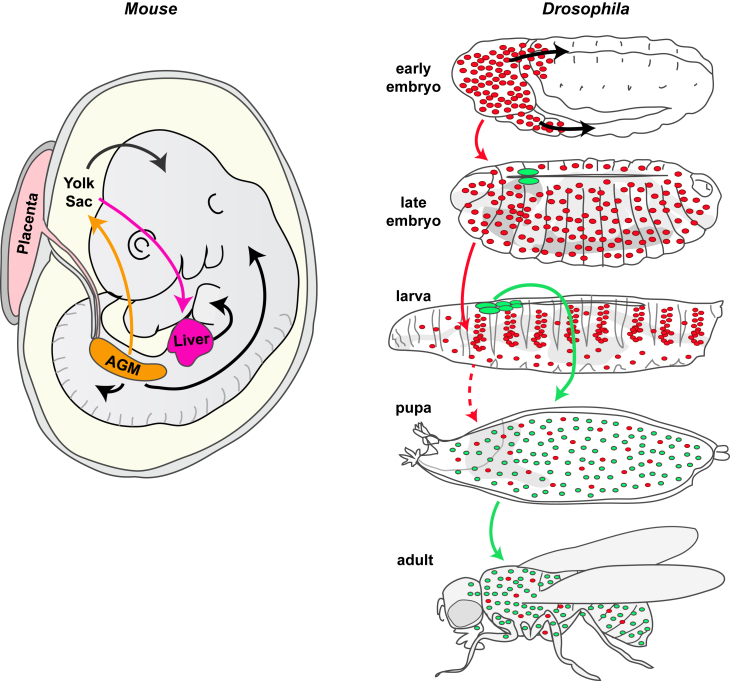
Hematopoiesis in Mouse and Fly A schematized, limb bud stage mouse embryo with arrows indicating the flow of macrophage progenitors, which are all initially derived from the yolk sac and aorta-gonad-mesonephros (AGM), but with some populations moving directly onto their eventual tissues and others bypassing and differentiating further in the liver. In *Drosophila* (right), as in vertebrates, hematopoiesis occurs in two waves. The first during early embryogenesis gives rise to embryonic macrophages (red) that disperse throughout the embryo and later populate the larva organizing into sessile patches and circulating blood cells; these can be considered the fly equivalent of tissue macrophages. A second population arise from the larval lymph gland (green); these cells are released during pupal development, make up most of the population of blood cells in both the pupa and the adult, and can be considered the fly equivalent of bone marrow-derived macrophages.

In the last 10 or so years, various tracking and lineage fate mapping studies in mice have made large inroads into discovering from where all the macrophage-like cells in various tissues are derived. GATA1/2 and PU.1 are key hematopoietic transcription factors that directly interact to repress alternative lineage programs and when PU.1 activity dominates, monocytes/macrophages develop (Chou et al., 2009). In large part it appears that successive waves of precursor monocytes, originating either from the yolk sac or the aortic endothelium, give rise to macrophage progenitors that either differentiate locally in the case of the yolk sac or migrate to the fetal liver, and go on to seed most embryonic tissues to give rise to the various tissue-resident macrophage populations. Surprisingly, for some tissues in particular, these resident cells are subsequently fairly stable and persist into adulthood, independent of bone marrow-derived contributions. There are still some controversies concerning precisely how some of the early tissue macrophage lineages are specified, but it seems clear that at least brain macrophages (microglia) arise directly from yolk sac-derived cells and turn over very little throughout life, whereas other tissues are subsequently replenished by contributions from fetal liver-derived monocytes. In the absence of trauma, this happens to different degrees such that some tissues receive only the lightest topping up by circulating bone marrow-derived monocytes (e.g., Langerhans cells of the epidermis, alveolar macrophages of the lung, and Kupffer cells of the liver), while others are slowly (e.g., macrophages in the heart) or rapidly (resident macrophages of gut and dermis) replenished by bone marrow-derived monocytes (reviewed in Ginhoux and Guilliams, 2016) (Figure 1). Part of the difficulty in deciphering which are the precise sources of macrophages in each of these tissues is that deleting one sublineage of an early precursor may result in compensatory expansion by another, and indeed it is likely that populations of macrophages are, in part, defined by their capacity to access each tissue and by competition between these precursors. Another difficulty is that the dynamic dispersal and migration of cells from their origins cannot be readily observed in real time in mammalian embryos.

### Developmental Dispersal of Macrophages Can Be Live Imaged in the Translucent Fly Embryo

**Figure 2:**
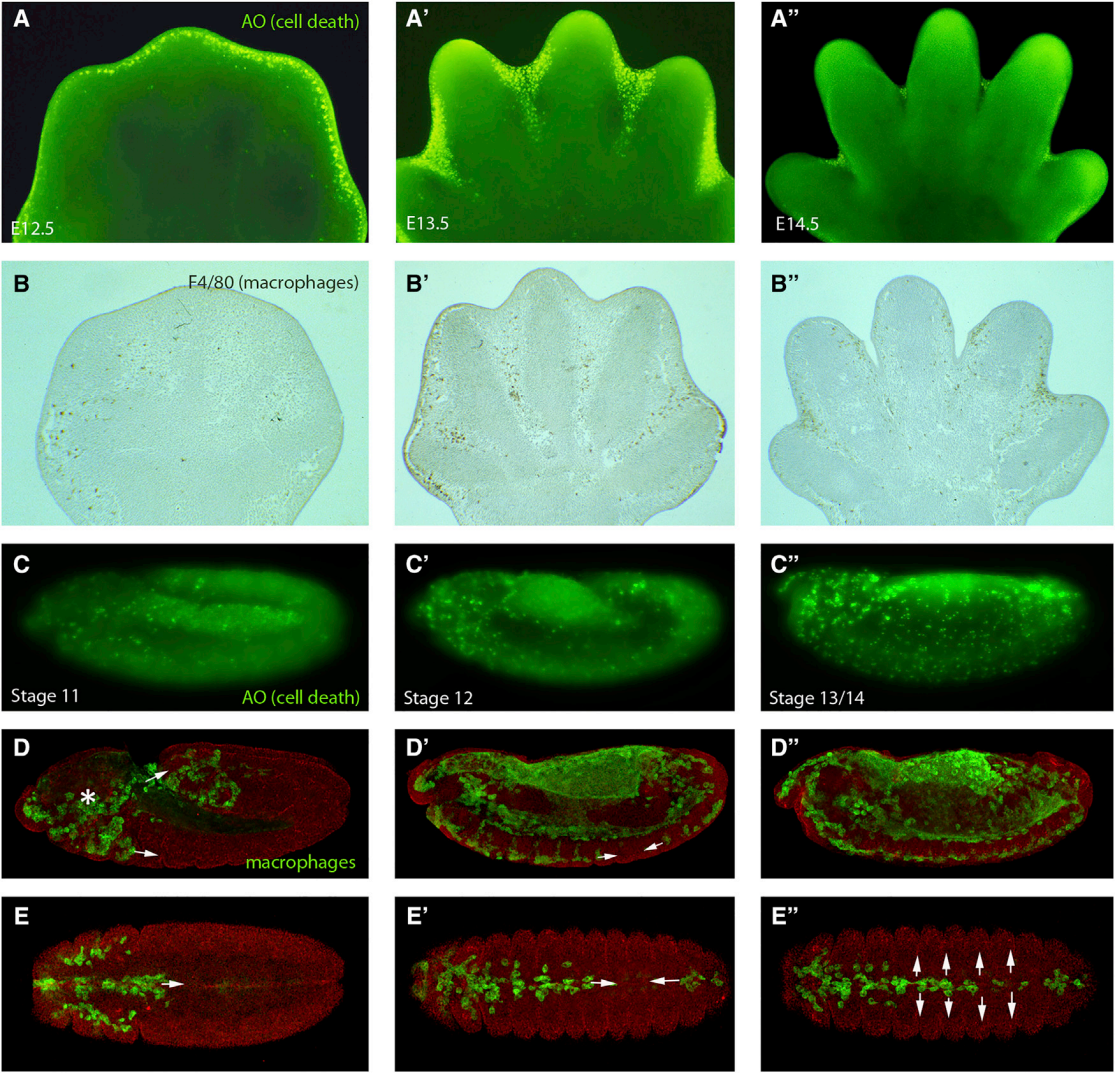
Macrophages Clear Developmental Apoptosis during Development in the Mouse and Fly Acridine orange (AO) staining of mouse embryo footplates between 12.5 and 14.5 days of development reveals cell death (bright green) in the interdigital tissue of the developing limb (A–A″). Corresponding stage limbs stained with F4/80 reveal macrophages (brown) in the same location as they engulf the resulting apoptotic corpses (B–B″). AO staining in the *Drosophila* embryo (bright green in C–C″) or expressing GFP in macrophages (green in D–D″) reveals a similarly tight correlation between position of developmental cell death and macrophages throughout development in the fly embryo. Fly macrophages are born in the head (asterisk in D) and migrate through two routes, one into the extended germband and one along the ventral midline (arrows in D). (E)–(E′) show ventral views of *Drosophila* embryos at stages corresponding to those in (C)–(C′), highlighting the developmental migration of macrophages (green) along the ventral midline (arrows in E′). This is then followed by a rapid lateral migration from the midline (arrows in E″).

Hematopoiesis has been well studied in the fly and the signaling that drives blood cell progenitor formation, maintenance, and differentiation appears to be fairly well conserved between *Drosophila* and mammals (reviewed in Crozatier and Vincent, 2011, Evans et al., 2003, Gold and Bruckner, 2014, Wood and Jacinto, 2007). Just as in vertebrates, the sites of hematopoiesis in the fly change as development proceeds (Figure 1). *Drosophila* hematopoiesis occurs in two waves. The first cohort of blood cells derive from head mesoderm of the developing embryo and give rise to both macrophages and crystal cells. These cells can be considered the fly equivalent of erythromyeloid progenitor (EMP)-derived tissue macrophages (Gold and Bruckner, 2015), and their specification requires similar molecular players to those that control mammalian hematopoiesis with the GATA factor Serpent (Srp) in combination with the friend of GATA (FOG) transcription factor U-shaped (Ush) operating as master regulators of blood cell fate (Fossett et al., 2001, Holz et al., 2003, Lebestky et al., 2000, Rehorn et al., 1996, Tepass et al., 1994, Waltzer et al., 2002). The proliferation and survival of these macrophages is then regulated by the fly orthologs of the vertebrate platelet-derived growth factor/vascular endothelial growth factor (PDGF/VEGF) family of growth factors (Pvf) (Bruckner et al., 2004, Sopko et al., 2015). After their birth, embryonic macrophages have to disperse from the head mesoderm to distribute themselves throughout the embryo such that at the end of embryogenesis they are evenly distributed throughout the animal (Tepass et al., 1994) and can actively engulf bacteria upon infection (Tan et al., 2014, Vlisidou and Wood, 2015). The translucency of fly embryos makes these developmental migrations very amenable to live imaging studies, unlike those of their vertebrate counterparts. Macrophage dispersal throughout the fly embryo is achieved through a developmentally hardwired pattern of migrations that are orchestrated, at least partly, by chemotactic signals provided by the Pvf family of growth factors (Cho et al., 2002, Wood et al., 2006). These migrations funnel macrophages along a number of specific routes: initially they migrate out from the head mesoderm and either infiltrate the extended germband or migrate along the developing CNS in the ventral midline of the developing embryo (Figure 2). Once they have populated the entire length of the developing CNS they spread laterally in a series of “rib-like” migrations that are, in part, patterned by the process of contact inhibition (Davis et al., 2012, Wood et al., 2006) (Figure 2). These developmental migrations involve exquisitely regulated reorganizations of the actin cytoskeleton to generate dynamic actin-rich protrusions, both lamellipodia and filopodia, which the cells use to power their migrations to all regions of the embryo. How macrophages assemble and regulate these protrusions in vivo is complex, with their dynamics depending on the combined action of many actomyosin regulatory proteins including the Rho family of small guanosine triphosphatases (GTPases), Rho, Rac, and Cdc42 (Paladi and Tepass, 2004, Stramer et al., 2005). Downstream of small GTPase signaling, the Vasp family member Enabled (Ena) plays a key role in directing lamellipodial protrusions (Tucker et al., 2011), and the actin bundling protein fascin is important for stabilization of these structures (Zanet et al., 2009). The Arp 2/3 activator SCAR/WAVE is also important for the formation of lamellipodia (Evans et al., 2013), and recent work has uncovered an intriguing crosstalk between Ena and the formin Diaphanous (Dia) in macrophages where Ena negatively regulates Dia to dictate which kind of protrusion is made (Bilancia et al., 2014).

The actin-rich lamellae provide the engine for motility, but directionality is dependent also on a bundled microtubule “compass” arm that also appears to enable contact inhibition of locomotion (CIL), which, in turn, is pivotal for equal dispersal of macrophages beneath the embryonic epidermis (Davis et al., 2012, Stramer et al., 2010). A recent paper has shed light on the mechanism by which this CIL process occurs, with the rapid repulsion from a neighboring cell being driven by the sudden release of tension that builds up at the interface between two colliding cells (Davis et al., 2015). How individual actin and microtubule regulatory proteins coordinate their action to control the dynamics, polarity, and nature of these protrusions in macrophages remains an area of intense interest and study.

A second wave of hematopoiesis in flies occurs post-embryonically in a specialist larval organ called the lymph gland (Figure 1). This organ supplies blood cells at the beginning of metamorphosis (Crozatier et al., 2007, Jung et al., 2005, Lanot et al., 2001) and gives rise to all three types of *Drosophila* blood cell: macrophages (plasmatocytes), crystal cells, and lamellocytes. These macrophages can be considered the fly equivalent of vertebrate bone marrow-derived macrophages (Gold and Bruckner, 2015), and studies have revealed a number of signaling pathways that play key roles in directing this hematopoietic program. A pool of progenitor blood cells is maintained within the larval lymph gland under the control of a posterior signaling center (PSC), which expresses the fly homolog of the vertebrate EBF-1 transcription factor, Collier (Krzemien et al., 2007). This signaling center operates as a stem cell niche to control blood cell homeostasis acting in a non-cell-autonomous manner to maintain the activity of the Hedgehog (Hh) and JAK-STAT pathways in the progenitor cells, which maintains their multipotency (Mandal et al., 2007). Wingless (Wg), the fly ortholog of vertebrate Wnt signaling, has also been shown to control the maintenance of hematopoietic progenitor cells within the lymph gland (Sinenko et al., 2009). The activity of the PSC niche in the fly can be modulated by physiological constraints reminiscent of the interactions described in vertebrates between hematopoietic stem cells and their microenvironment. A key study established reactive oxygen species (ROS) as a regulator of fly hematopoiesis by revealing that ROS levels in progenitor cells sensitize these progenitors to differentiate (Owusu-Ansah and Banerjee, 2009). The maintenance of hematopoietic progenitor cells can also be directly influenced by the nutritional state of the fly as well as by levels of sensory perception in the animal (Shim et al., 2012, Shim et al., 2013).

Recent studies have focused on the period of larval development between these two phases when hematopoiesis is initiated through the colonization of hematopoietic microenvironments by existing blood cells (reviewed in Makhijani and Bruckner, 2012) (Figure 1). Clues as to the signals that might regulate hemocyte survival and differentiation come from studies showing that colonization of these hematopoietic pockets is driven by attractive and trophic cues from neurons of the peripheral nervous system (Makhijani et al., 2011) and requires epidermal growth factor (EGF)-like receptor signaling (Bretscher et al., 2015). Once at these sites cells divide at a higher rate and are able to undergo transdifferentiation into crystal cells (Leitao and Sucena, 2015). There are clear parallels here with mammals, since in the vertebrate bone marrow sympathetic nerves and their associated glia regulate hematopoietic stem cell localization, proliferation, and maintenance (Katayama et al., 2006, Mendez-Ferrer et al., 2008, Mendez-Ferrer et al., 2010, Spiegel et al., 2007, Yamazaki et al., 2011). Further genetic investigations in the fly will provide more valuable insight into how local microenvironments can regulate self-renewing tissue macrophages.

### How Macrophages Sculpt and Pattern Mammalian Embryonic Tissues

During vertebrate embryonic development, aside from seeding tissues with cells that will provide a surveillance function against microbial invaders and the capacity to raise a local and systemic inflammatory response, several other roles for macrophages have been uncovered. Their best known role is as a scavenger of apoptotic corpses that arise during development. The developing nervous system, for example, gives birth to many more neurons than will be successfully integrated into the developing brain and spinal cord, and the unnecessary cells die through lack of neurotrophic support; early in the apoptotic process they are recognized by macrophages and engulfed. The extent of this apoptosis (almost half of all neurons that are born), and their clearance, was initially missed because both the death and clearance events are relatively rapid (and so appear rare), by comparison with the period over which this neural remodeling occurs (Raff et al., 1993). More immediately dramatic are events within tissues that are sculpted by synchronized aggregations of local cell death, as for example in the interdigit regions of mouse embryo footplates leading to digit separation, and in these situations macrophages are drawn in large numbers, with each able to engulf several apoptotic cells and clear tissues of corpses within hours (Figure 2; Wood et al., 2000). We know that professional phagocytic lineages are not essential per se for clearing apoptosis because of corpse clearance in organisms such as *Caenorhabditis elegans*, where no professional phagocytic lineage exists, and indeed in murine embryos null for the lineage-switching ETS-family transcription factor, PU.1, which lack all macrophages, where it seems that “amateur” phagocytes, in the form of local tissue fibroblasts, can stand in but are less efficient in clearing away the corpses (Wood et al., 2000). In both the trickle cell death, as occurs in the nervous system, and synchronized apoptosis scenarios, like in the footplate, macrophages do not direct the killing themselves but rather respond to and clear it away. However, there are situations where they do provide positive killing signals; for example, if macrophages are depleted in the developing rodent eye, a network of capillaries that would normally regress through endothelial cell apoptosis instead persist (Diez-Roux and Lang, 1997); this killing signal from macrophages is now known to be Wnt 7b (Lobov et al., 2005).

### Fly Genetics and Live Imaging Opportunities Have Enabled a Detailed Dissection of the Engulfment Signaling Machinery

**Figure 3:**
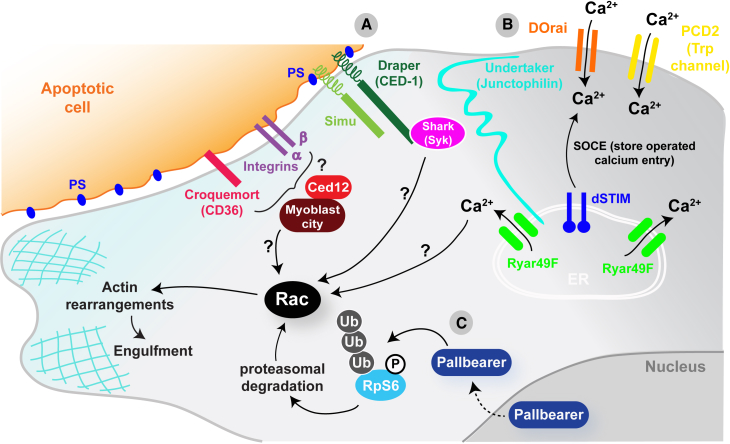
Apoptotic Recognition and Clearance Signaling in the Fly Several transmembrane proteins have been identified that allow detection of apoptotic corpses in the fly (A) including Croquemort (homolog of vertebrate CD36 scavenger receptor), the CED-1 homolog Draper, Six-microns under (Simu), and βv/αPS3 integrin heterodimers. Draper and Simu have been shown to bind phosphatidylserine (PS) on the surface of apoptotic cells, but how activation of any of these receptors upon binding to their ligands leads to the activation of Rac and the subsequent actin rearrangements required for engulfment remains largely unknown. In the case of Draper, activation of Rac could be through the Syk kinase homolog shark and in other cases will likely involve ELMO/Ced12 and Myoblast city. Two signaling cascades have been identified in the fly macrophage downstream of apoptotic engagement. The first (B) involves a calcium signaling pathway driven by intracellular store operated calcium entry (SOCE) downstream of Draper. A junctophilin (undertaker), an ER calcium sensor (Dstim), a calcium release activated channel (DOrai), and a TRP channel (Pkd2) are all required for this calcium signaling event. Again, how this calcium signaling leads to the activation of Rac remains unknown. The second (C) involves an F-box protein that acts as an E3 ubiquitin ligase called Pallbearer. This interacts with phosphorylated ribosomal protein S6 (RpS6) to promote its ubiquitylation and proteasomal degradation, which leads to Rac activation and subsequent actin remodeling required for engulfment.

Like their vertebrate counterparts, *Drosophila* macrophages function as professional phagocytes within the embryo, efficiently engulfing and degrading large numbers of apoptotic corpses generated during normal development. How macrophages detect, engulf, and degrade apoptotic corpses is an intensely studied field, and once again there appears to be strong conservation of molecular mechanism from the fly to vertebrates. *Drosophila* macrophages use a battery of receptors including, among others, croquemort, a homolog of the vertebrate CD36 scavenger receptor (Franc et al., 1996), the CED-1 homolog Draper (Manaka et al., 2004), and βv/αPS3 integrin heterodimers (Nonaka et al., 2013), to recognize “eat me” epitopes such as phosphatidylserine (PS) on their apoptotic prey (Tung et al., 2013). In mammals redundancy among phagocytic receptors is higher, and in many cases loss of a single receptor function does not result in abnormal apoptotic cell clearance. Live imaging and significantly less redundancy have allowed the fly to emerge as a powerful system to dissect the machinery required for apoptotic clearance. These studies have uncovered several new important players in the process, including Six-Microns-Under (SIMU, also known as Nimrod C4), a transmembrane tethering receptor that is also able to act as a secreted bridging molecule binding PS on apoptotic corpses (Kurant et al., 2008, Shklyar et al., 2013), and Pretaporter, an intracellular protein that can operate as an “eat me” signal on apoptotic cells when translocated to the plasma membrane (Kuraishi et al., 2009). The fly has also provided insights into the signaling events occurring within macrophages downstream of apoptotic engagement and has uncovered a pair of signaling cascades. The first involves an F-box protein that acts as an E3 ubiquitin ligase called Pallbearer, operating in an SCF (Skp Cullin F box) complex (Xiao et al., 2015), and the second a calcium signaling pathway driven by intracellular store operated calcium entry (SOCE) downstream of Draper (Cuttell et al., 2008). Genetic studies have also identified a junctophilin (undertaker), an ER calcium sensor (Dstim), a calcium release activated channel (DOrai), and a TRP channel (Pkd2) that are all required for this critical calcium signaling event (Cuttell et al., 2008). The fly homolog of Ced-12 (dCed-12), which is also involved in apoptotic phagocytosis, has been shown to function in a parallel genetic pathway analogous to its *C. elegans* homolog (Van Goethem et al., 2012) (Figure 3).

### More Than Just Killers and Eaters

While macrophages are clearly best known for their capacity to phagocytose corpses, they have several other developmental patterning roles that are not directly linked to apoptosis or phagocytosis. These functions come to light in mice null for transcription factors that are key for macrophage differentiation, for example, PU.1 and Csf1. Such mice have defects in organs where branching morphogenesis is pivotal, for example the lung and kidney, and in the mammary gland, where this link has been most closely studied, there is some evidence to suggest that appropriate branching might be mediated by regulation of the degree and pattern of collagen deposition around the bud sprouts (Ingman et al., 2006). Regulation of matrix deposition, alongside local delivery of angiogenic factors such as VEGF, may also explain the role that macrophages play in several aspects of developmental angiogenesis and lymphangiogenesis, whereby macrophages have been observed wrapped around and apparently nurturing vessel sprouts as tip cell fusion leads to vessel anastomosis in the developing mouse and zebrafish brains (Fantin et al., 2010).

Macrophages also play key roles in establishing “niches” that allow other cell lineages to flourish. For example, in the pancreas, clusters of macrophages provide the microenvironment that enables islet cell development, and loss of macrophages, as in the Csf1 KO mouse, results in far fewer islet producing B cells, whereas addition of macrophages to pancreatic organ culture increases B cell numbers (Banaei-Bouchareb et al., 2006, Geutskens et al., 2005). There is good evidence that macrophages are important in maintaining stem cell niches in both the colon and mammary gland (Gyorki et al., 2009, Pull et al., 2005), and in both male and female gonad it seems that macrophages may also be critical. In the ovary, follicle rupture through the ovary wall to release eggs is dependent on macrophages (Brannstrom et al., 1993, Pollard, 2009), and in the testis macrophages line the surface of seminiferous tubules where undifferentiated spermatogonia lie, and appear to directly regulate spermatogonial differentiation via release of factors including Csf-1 and retinoic acid biosynthesis enzymes (DeFalco et al., 2015).

Similarly, in the fly, macrophages play many “patterning” roles during development and their correct distribution around the embryo is critical for various subtle aspects of organogenesis. As described earlier, one early migratory route for *Drosophila* macrophages is along the developing embryonic ventral nerve cord (Figure 2) where there is a clear interdependence between macrophage migration and correct CNS development (Evans et al., 2010). A loss of macrophages leads to a failure in CNS condensation and miswiring of the nervous system (Olofsson and Page, 2005, Sears et al., 2003). Another migratory route guides macrophages across the yolk sac and into the extended germband (Bruckner et al., 2004, Cho et al., 2002). Here the macrophages must become invasive and breach the tissue barrier presented by the germband epithelium (Siekhaus et al., 2010). This penetrative migration is dependent on integrin function regulated by the GTPase Rap1 (Siekhaus et al., 2010) in ways that mirror the transepithelial migration of vertebrate neutrophils and monocytes out of the vasculature and toward sites of inflammation (Abram and Lowell, 2009). Once inside the germband, some of these macrophages then come into contact with the fly equivalent of the developing kidney, the Malpighian tubules, where again they play a key role in influencing organogenesis by secreting collagen IV, which is required for effective bone morphogenetic protein (BMP) signaling that in turn directs the outgrowth and positioning of these organs (Bunt et al., 2010). These clear parallels lay the foundations for researchers to take advantage of the powerful genetics and live imaging opportunities in the fly to inform vertebrate studies as to how macrophages might influence the development of many tissues within the embryo.

### *Drosophila* Macrophages and Their Capacity to Clear Infections

As well as developmental roles fly macrophages, like their vertebrate counterparts (macrophages and, to a larger extent, neutrophils), play an important sentinel role in the immune system to protect the individual against invading pathogens. Studies in the fly using larval macrophages ex vivo to interrogate the phagocytic machinery required for internalization of bacteria revealed the Nimrod family of receptors, Eater (Kocks et al., 2005) and NimC1 (Kurucz et al., 2007), as being important for the recognition and uptake of Gram-positive and Gram-negative bacteria. Draper has been shown to mediate the uptake of *Staphylococcus aureus* in adult flies (Hashimoto et al., 2009), and a recent study has demonstrated that Rab14 is essential for phagosome maturation following engulfment of the same bacterium (Garg and Wu, 2014). Studies using *Drosophila* macrophage-like S2 cells have identified other phagocytic receptors such as the scavenger receptor Peste, which is required for the uptake of *Mycobacterium fortuitum* but not *Escherichia coli* or *S. aureus* (Philips et al., 2005), dSR-C1 that recognizes both Gram-positive and Gram-negative bacteria, and the peptidoglycan recognition protein LC (PGRP-LC), which mediates the uptake of *E. coli* (Ramet et al., 2002).

Following infection in the fly, macrophages do not act exclusively as phagocytic cells to clear the invading microorganism but also carry out signaling roles to coordinate systemic immune responses across different tissues. In part this role is needed because of the absence of an adaptive immune response in flies. For example, septic injury to adult flies has been shown to induce the production of the cytokine Unpaired 3 (Upd3) in macrophages, which then activates JAK/STAT signaling in the fly equivalent of the liver, the fat body (Agaisse et al., 2003). Following gut infection with the phytopathogen *Erwinia carotovora* (Ecc15), macrophages are required for the induction of the expression of the antimicrobial peptide, Diptericin, in the fat body (Basset et al., 2000), and macrophages have been shown to relay Ecc15 infection-induced oxidative stress signals in the gut to the fat body to trigger antimicrobial peptide production (Wu et al., 2012). Expression of another antimicrobial peptide, defensin, in the fat body has been shown to be dependent on pathogen degradation within macrophages via the lysosomal protein Psidin (Brennan et al., 2007), and antimicrobial peptide production in the fat body following septic injury has also been shown to require a signal relayed by macrophages through secretion of the Toll pathway ligand Spatzle (Shia et al., 2009).

Infection by bacteria is not the only immune threat faced by the fly. In the *Drosophila* larva infestation by parasitoid wasps, such as *Leptopilina boulardi*, has been studied extensively and provides a fascinating model for studying macrophage immune behavior in vivo. Upon parasitization, macrophages rapidly mobilize and differentiate into a specialist cell known as a lamellocyte, which forms a multilayer capsule around the parasitic wasp egg in cooperation with macrophages and a third blood cell type, crystal cells (Markus et al., 2009). Following this initial response, blood cells of the lymph gland undergo a proliferative burst and differentiate into lamellocytes, which are released into circulation (Lanot et al., 2001, Rizki and Rizki, 1992, Sorrentino et al., 2002), a process that requires the steroid hormone ecdysone and signaling from the PSC of the lymph gland (Benmimoun et al., 2015, Crozatier et al., 2004). A recent study has shown that macrophages can transdifferentiate into lamellocyte-like cells in situ directly on the wasp egg (Anderl et al., 2016). Perhaps the closest parallel with this behavior of macrophages in *Drosophila* is the granuloma response in vertebrates to *Mycobacterium tuberculosis* infection whereby infected macrophages are “walled off” by layers of uninfected macrophages that fuse and form an epithelial-like barrier to contain the infection (Cronan et al., 2016, Pagan and Ramakrishnan, 2014).

### Responding to Inflammatory Signals in Damaged or Altered Mammalian Tissues

**Figure 4:**
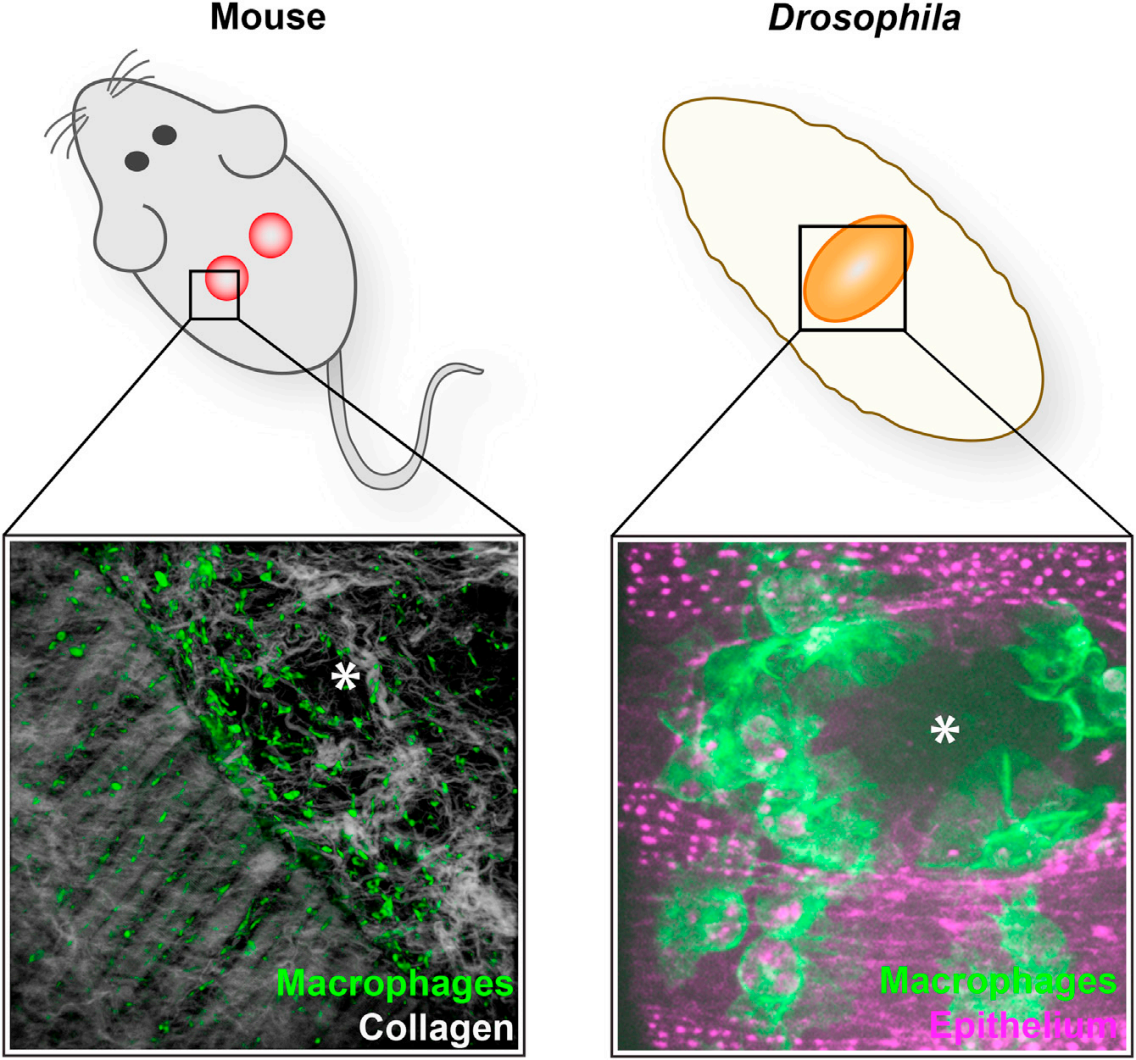
Wounding Triggers a Recruitment of Macrophages in the Mouse and Fly Right: F480 immunostaining of a wound made to the back skin of an adult mouse with multiphoton second harmonics revealing collagen (white) to reveal the wound margin running from top left to bottom right of the field of view. Macrophages (green) are clustered at the wound edge. Left: similarly, laser ablation wounds made in the epithelium of a fly embryo trigger a rapid chemotactic response from macrophages (green), which are recruited to the wound within minutes and remain at the wound site throughout closure. Wounds are marked with an asterisk. Mouse wound image courtesy of Jenna Cash, and fly image courtesy of Helen Weavers.

Innate immunity is clearly critical following any wounding episode to prevent septicemia as opportunistic microbes enter gaps where the barrier layer is breached. After tissue damage in mammals, macrophages tend to follow in the wake of neutrophils and actively accumulate at the wound site, deriving from two sources, tissue-resident macrophages that are already in the vicinity of the wound and recruited monocytes that are drawn from the local wound vasculature (Shaw and Martin, 2016) (Figure 4). At the wound site macrophages fulfill a portfolio of roles that change during the time of healing; initially they are bactericidal, as well as voraciously phagocytosing cell and matrix debris, particularly clearing red blood cells and spent neutrophils at the wound site. At later times they develop pro-repair capacity, for example promoting wound angiogenesis through the release of Vegf and other angiogenic factors. These changing phenotypic roles may be primed by previous experiences, and are believed to reflect altered macrophage polarities, from resting, M0, through to bactericidal, M1, and subsequent various M2a, b, c, and d states (Crane et al., 2014, Dal-Secco et al., 2015), but whether these changes occur in individual macrophages or are partly a consequence of successive incoming waves of cells with different activities is still unclear.

Macrophages are not absolutely critical for mammalian healing per se, because embryonic tissues can repair at stages before the first macrophages are born, and neonatal mice null for PU.1 that have no macrophages can repair wounds very efficiently; indeed, they do this without leaving any trace of a scar, just as in the embryo, which is suggestive that macrophages mediate wound fibrosis (Martin et al., 2003). However, adult tissue repair appears much more dependent on macrophages, with classic antimacrophage serum knockdown experiments in rabbits exhibiting poor healing (Leibovich and Ross, 1975), and more recent temporally regulated diphtheria toxin killing of macrophages in mice revealing different healing defects depending on which phase of healing is targeted: early knockdown of macrophages results in retarded re-epithelialization and reduces the extent of wound granulation tissue and eventual scar size, whereas mid-stage knockdown leads to a failure of granulation tissue maturation and contraction and to severe wound hemorrhaging, suggesting that macrophages may be orchestrating key behaviors at different times and in several cell lineages within the healing wound (Lucas et al., 2010).

Changes in macrophage phenotype/plasticity during the wound inflammatory response may be pivotal in how they interact with the wound cells sharing their environment. There have long been hints that tissue scarring is evolutionarily linked to the type-2-cell mediated immune response to parasitic infections that lead to fibrous encapsulation of helminths as a host protection response (Allen and Sutherland, 2014). It is believed that just as macrophage phenotype switching via IL4R activation drives parasitic encapsulation, it might also lead to tissue scarring, and a recent study shows that this is mediated by Relm-α signaling which, in turn, drives expression of persistent collagen crosslinking enzymes leading to the bundled unresolvable collagen of a dermal scar (Knipper et al., 2015).

### *Drosophila* Offers Insights into the Earliest Damage Cues that Draw Macrophages to Wounds

**Figure 5:**
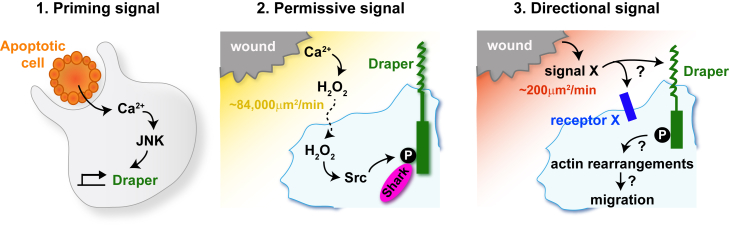
A Three-Part Signaling System Drives the Inflammatory Response in the Fly (1) In *Drosophila*, macrophages are initially primed to respond to a wound by engulfing an apoptotic corpse. The process of engulfment triggers a calcium signaling event in the macrophage which, through activation of the JNK pathway, leads to upregulation of the damage receptor draper and makes these cells “primed” for response to a subsequent wound. (2) Upon wounding, hydrogen peroxide (H_2_O_2_) is rapidly released from the wound site diffusing at approximately 84,000 μm/min, acting as a “permissive signal” for macrophage migration to wounds by activating Src-dependent phosphorylation of Draper on its ITAM domain, which in turn recruits the downstream kinase shark. (3) A third unknown directional signal (signal X) is also produced upon wounding and diffuses away from the wound at a speed of approximately 200 μm/min. This signal operates as an attractive cue to pull the macrophage to the wound and could be detected by Draper or unknown damage receptors (receptor X).

In recent years genetic and live imaging studies in *Drosophila* have provided important insights into the earliest events that allow macrophages to detect, and be recruited to, sites of damage or altered cell states. The best characterized of these damage responses is the rapid inflammatory-like chemotactic response of macrophages toward wounds in the embryo (Evans and Wood, 2014, Stramer et al., 2005) (Figure 4). We now know a considerable amount about the immediate signaling that triggers recruitment of macrophages to a wound in the fly, and once again the mechanisms appear to show strong conservation through to vertebrates. In worms, flies, and fish, wounding induces a rapid calcium flash that spreads across the wounded epithelium as a wave (Antunes et al., 2013, Razzell et al., 2013, Xu and Chisholm, 2011, Yoo et al., 2012). In *Drosophila* this calcium signal activates the NADPH oxidase Duox within the epithelium to generate hydrogen peroxide (H_2_O_2_) at the wound (Razzell et al., 2013), which operates as an early damage signal required for the recruitment of blood cells to wounds in both the fly and fish (Moreira et al., 2010, Niethammer et al., 2009). Studies in zebrafish identified the redox-sensitive Src family kinase (SFK), Lyn, as being critical for leukocytes to detect and respond to damage-induced H_2_O_2_ (Yoo et al., 2011), and a recent study in *Drosophila* has shown the same requirement for the fly homolog of Lyn, Src42A, during macrophage recruitment to wounds (Evans et al., 2015). The same study further showed that the fly equivalent of the vertebrate immune SFK-ITAM-domain-Syk signaling pathway involved in vertebrate adaptive immunity plays a key role in macrophage recruitment by wound-induced H_2_O_2_ (Evans et al., 2015) (Figure 5). Small GTPase molecular switches are needed for this migration, with Rac and Rho enabling assembly of leading-edge lamellipodia and retraction of the trailing tail, respectively, while Cdc42 is needed for polarized migration to the wound (Stramer et al., 2005). Curiously, while developmental dispersal migrations are independent of phosphatidylinositol 3-kinase signaling, this pathway is vital for responsiveness to a wound (Wood et al., 2006), although how this signaling is linked to the coordination of the actin cytoskeleton remains unknown.

At the end of *Drosophila* embryogenesis, the primitive fly heart begins to beat and macrophages are then pumped around the extracellular space within the larva. These circulating larval macrophages can be passively captured at sites of wounding by a process that resembles the rolling and tethering of vertebrate leukocytes that occurs before extravasation from vertebrate wound vessels, although it clearly does not model later aspects of extravasation through the vessel wall (Babcock et al., 2008). Later, in pupal life, hemocytes regain their capacity for active migration to sites of tissue damage and large numbers are drawn to wounds made in pupal tissues. Since wounds in the pupae can be bigger and the inflammatory response therefore involves a larger number of macrophages, this stage has been best for generating large amounts of tracking data and thus has enabled mathematical modeling studies to be carried out, providing new insight into inflammatory cell response to damage cues (discussed later).

### Macrophages Provide a Taxi Service for Mammalian Cancer Cells as They Begin to Metastasize

Both the innate and adaptive immune systems are known to play a role in cancer surveillance but also in cancer progression, and it is clear from patient studies that the presence and phenotypic state of macrophages within different cancer types can significantly alter prognostic outcome (Noy and Pollard, 2014). Mechanistic studies of how macrophages influence cancer progression is difficult in mouse because opaque tissues make imaging difficult, but intravital imaging studies of xenografted cancer cells within the mammary fat pad have shown a clear involvement of macrophages in the initial step of metastasis, where they help shuttle cancer cells from the primary tumor to nearby vessels, from which they can then spread to secondary sites; these studies have revealed a mutually supportive paracrine loop with cancer cell synthesized CSF-1 and macrophage-derived EGF together guiding the directional movement of both cells toward local vessels (Condeelis and Pollard, 2006, Wyckoff et al., 2007). Studies of the early cancer initiation stages when pre-neoplastic cells are first born in tissues are easier in the translucent zebrafish, where it seems that neutrophils and macrophages rapidly detect these abnormal cells and may nurture them by providing trophic signals (Feng and Martin, 2015, Feng et al., 2010, Freisinger and Huttenlocher, 2014).

### *Drosophila* as a Model to Study Immune Cell Responses to Cancer

As in vertebrate tissues, fly macrophages are readily recruited to and can influence abnormally growing clones of cells. Tumors induced by expression of oncogenic Ras^V12^ or by mutations in the polarity genes scribble, discs large, or lethal giant larvae lead to the attraction and adhesion of macrophages to the mutant tissue (Cordero et al., 2010, Hauling et al., 2014, Pastor-Pareja et al., 2008; reviewed in Ratheesh et al., 2015). In polarity gene mutation-mediated tumors, macrophages inhibit tumor growth via the production of the *Drosophila* tumor necrosis factor (TNF) ortholog, Eiger (Parisi et al., 2014). However, if these tumors also express Ras^V12^ the tumor cells hijack this macrophage response for their own gain such that macrophage-secreted TNF-α leads to tumor overgrowth and invasion (Cordero et al., 2010). This is analogous to vertebrate tumor-associated macrophages promoting tumor function and pro-inflammatory cytokine production through TNF-α signaling (Ostuni et al., 2015). Interestingly, a recent study has identified a role for macrophages in triggering apoptosis-induced proliferation (AiP), a process whereby caspase-initiated signaling cascades in apoptotic cells leads to the proliferation of neighboring cells. In this study the authors showed that macrophages are recruited to sites of AiP by Duox-triggered ROS where they activate JNK signaling in epithelial cells by production of Eiger (Fogarty et al., 2016). This work reveals an intriguing signaling axis between macrophages and epithelial cells, which may shed further light on how macrophages drive epithelial growth and the related tumor-promoting role of inflammation.

### Where Else Might Flies Offer Insights into Functions and Signaling Machinery in Macrophages?

One fascinating aspect of macrophage biology that can perhaps be best studied in the fly is that of signal integration and prioritization. For a macrophage to efficiently migrate toward a given target it must have the capacity to detect the end-target attractant along with other intermediate cues en route, while integrating these signals with other potentially distracting ones within its environment and prioritizing appropriately to prevent being pulled in disparate directions. This remarkable capacity for navigation using several cues over relatively large distances has been partially studied in vertebrate leukocytes through elegant in vitro approaches (Foxman et al., 1997, Foxman et al., 1999), but the fly offers opportunities for better understanding of this complex process in vivo. Studies have revealed that *Drosophila* macrophages can integrate competing signals in the embryo and exhibit hierarchical responses; for example, they will actively prioritize the Pvf growth factor cues that direct their developmental migrations over those attractant signals released by a wound, and will prioritize an apoptotic corpse over the developmental PVF tracks (Moreira et al., 2010). Because studies of fly wound inflammation enable live imaging and the collection of large datasets, particularly in pupae, it is now possible to use mathematical modeling to further investigate macrophage behaviors upon wounding and extrapolate more about the characteristics of the wound attractants from these behaviors. For example, simulations that approximate the real mean behaviors of macrophages responding to a wound indicate that the attractant diffuses at approximately 200 μm^2^/min, which is considerably slower than the diffusion coefficient for damage-associated molecular patterns (DAMPs) such as ATP and H_2_O_2_, suggesting that these signals can only be permissive factors and that the true attractant is a larger molecule (Figure 5). Another clue as to the nature of the attractant comes from modeling how two wounds might compete in recruitment of macrophages; if a second wound is made nearby but only 90 min after the first, macrophages in the vicinity are refractile to the second wound, but another 90 min later they regain responsiveness, and this period of desensitization is very reminiscent of a signal operating via G-protein-coupled receptors (Weavers et al., 2016b).

Another aspect of immune cell signal integration where the fly has recently provided a significant advance in our understanding is the process of innate immune priming or “trained immunity.” Emerging evidence from vertebrate studies has demonstrated that innate immune cells can develop a form of immunological memory, a trait previously associated only with the adaptive system (reviewed in Netea et al., 2016). A recent study in the fly has revealed the existence of this innate immune memory in *Drosophila*, where the phagocytosis of apoptotic cells by macrophages is an essential primer for their subsequent inflammatory response to tissue damage and infection (Weavers et al., 2016a). This study shows that before phagocytosing an apoptotic corpse, macrophages are naive and incapable of sensing wound signals or microbes, but upon their first corpse engulfment they exhibit a calcium flash that triggers a JNK-mediated upregulation of the CED-1 homolog Draper, which appears to drive a mid- to long-term priming to enable responsiveness to wounds and infections (Figure 5). This mechanism whereby macrophages change the levels of pathogen-associated molecular pattern and DAMP receptors on their surface to build a memory of previous encounters and reshape their response to subsequent insults is likely to be conserved across phyla and be pivotal in macrophage behavior in pathological scenarios.

Another study, this time using adult flies, has shown that macrophages become stimulated by neuronal injury and accumulate around degenerating distal axons in the wing (Soares et al., 2014), demonstrating that the fly may offer an attractive model for studying the immune response to neuronal damage and degeneration/regeneration. Besides the wound response, *Drosophila* have revealed other novel roles for macrophages. For example, a recent study uncovered an important physiological role for macrophages in regulating the fly's response to dietary stress. Flies fed a lipid-rich diet display reduced insulin sensitivity and life-span, and both of these effects are mediated by macrophages (Woodcock et al., 2015). This ability to control insulin signaling has clear parallels with vertebrates, where macrophages are critical for maintaining insulin sensitivity in adipocytes (Odegaard and Chawla, 2013) and where diseases associated with lipid-rich diets lead to activation of macrophages and the disruption of homeostasis (Biswas and Mantovani, 2012, Jin and Flavell, 2013, Moore and Tabas, 2011). Recent studies have also revealed a role for fly macrophages in maintaining and controlling the microenvironment of various stem cell niches. Macrophages have been shown to be required for the production of collagen IV in the basement membrane around the ovarian germline stem cell niche, and a loss of macrophages leads to abnormal adult niches with excess stem cells (Van De Bor et al., 2015). Another fascinating relationship between macrophages and stem cells was uncovered in a recent report where intestinal stem cells (ISCs) were shown to be regulated by macrophages during the early phase of intestinal regeneration in the fly. Upon damage to the intestinal epithelium, macrophages are recruited to the site of damage and secrete the fly ortholog of BMP, triggering ISC proliferation (Ayyaz et al., 2015). Another recent study showed that macrophages are able to remotely stimulate intestinal stem cell proliferation following septic injury via the production of the cytokine-like secreted proteins *Unpaired 2* and *Unpaired 3* (Chakrabarti et al., 2016). These studies pave the way for the fly to emerge as a powerful system to study how stem cell activity is coordinated with immune cell behavior as a consequence of an inflammatory response.

### Summary

Undoubtedly, not all that we learn from studies of macrophage signaling and function in flies will directly extrapolate to what the mammalian macrophage is doing in health and disease, but studies harnessing the live imaging opportunities in fly embryos, larvae, and now pupae provide a powerful model for the study of many aspects of macrophage biology, from the specification and developmental organization of these multitasking innate immune cells through to their many and varied roles at sites of disease. The powerful genetics of the fly will continue to inform vertebrate studies, and the integration of work in both systems will help provide a global picture of how these important therapeutic target cells function in both development and disease in the complex setting of a living organism.
